# Background Colonic ^18^F-Fluoro-2-Deoxy-D-Glucose Uptake on Positron Emission Tomography Is Associated with the Presence of Colorectal Adenoma

**DOI:** 10.1371/journal.pone.0160886

**Published:** 2016-08-10

**Authors:** Ko Eun Lee, Chang Mo Moon, Hai-Jeon Yoon, Bom Sahn Kim, Ji Young Chang, Hyo Moon Son, Min Sun Ryu, Seong-Eun Kim, Ki-Nam Shim, Hye-Kyung Jung, Sung-Ae Jung

**Affiliations:** 1 Department of Internal Medicine, School of Medicine, Ewha Womans University, Seoul, Republic of Korea; 2 Department of Nuclear Medicine, School of Medicine, Ewha Womans University, Seoul, Republic of Korea; Kyungpook National University School of Medicine, REPUBLIC OF KOREA

## Abstract

^18^F-fluoro-2-deoxy-D-glucose (FDG) positron emission tomography (PET) scan is used to evaluate various kinds of tumors. While most studies on PET findings of the colon focus on the colonic uptake pattern, studies regarding background colonic uptake on PET scan are rare. The purpose of this study was to identify the association between the background colonic uptake and the presence of colorectal adenoma (CRA), which is a frequent precancerous lesion. We retrospectively reviewed the medical records of 241 patients with gynecologic malignancy who had received PET or PET/computed tomography (CT) scan and colonoscopy at the same period as a baseline evaluation. Background colonic ^18^F-FDG uptake was visually graded and the maximal standardized uptake values (SUV_*max*_) of 7 different bowel segments were averaged. In univariate analysis, older age at diagnosis (≥ 50 years, *p* = 0.034), overweight (BMI ≥ 23 kg/m², *p* = 0.010), hypercholesterolemia (≥ 200 mg/dL, *p* = 0.027), and high grade background colonic uptake (*p* = 0.009) were positively associated with the prevalence of CRA. By multiple logistic regression, high grade background colonic uptake was independently predictive of CRA (odds ratio = 2.25, *p* = 0.021). The proportion of CRA patients significantly increased as background colonic uptake grade increased from 1 to 4 (trend *p* = 0.015). Out of the 138 patients who underwent PET/CT, the proportion of CRA patients in the group with high SUV_*max*_ (> 2.25) was significantly higher than in the low SUV_*max*_ group (27.5% vs. 11.6%, *p* = 0.031). In conclusion, high grade of background colonic ^18^F-FDG uptake is significantly associated with the prevalence of CRA.

## Introduction

^18^F-fluoro-2-deoxy-D-glucose (FDG) positron emission tomography (PET) scan is a functional imaging modality using the characteristics of FDG, which is accumulated more in tissues with increased glycolysis than in normal tissues. This is conceptually different from conventional structural imaging methods [1].

^18^F-FDG-PET is used in diagnosing various kinds of tumor, assessing tumor stage, and evaluating the treatment response [1]. In real clinical practice, baseline staging examinations for most kinds of cancer usually do not include colonoscopic evaluation, and some patients with gastrointestinal symptoms or possibility of colonic lesion in the radiographic imaging tend to undergo an additional colonoscopy. In colon, many studies focus on the FDG uptake pattern [1,2]. FDG uptake is classified into three patterns: focal, segmental, and diffuse. It is reported that focal uptake pattern is frequently associated with neoplasm such as colorectal adenoma (CRA) or colorectal cancer (CRC), and the segmental uptake pattern is more likely to be found in colonic inflammation such as colitis or inflammatory bowel disease [3–6]. Diffuse uptake pattern is usually considered as physiologic uptake [3,5,6].

To our knowledge, there have been few studies regarding background colonic uptake on PET. Underlying pathophysiology, related medical conditions, and clinical significance remain unknown. Recently, some studies reported that factors such as intestinal smooth muscle uptake, stool uptake, mucosal uptake, and lymphoid tissue uptake may affect physiologic intestinal ^18^F-FDG uptake [3,7–9]. In addition, the hypothesis that luminal bacteria and dyslipidemia affect background intestinal ^18^F-FDG uptake has been raised recently [10,11].

Therefore, we aimed to identify the clinical significance of background colonic ^18^F-FDG uptake on PET scan in real practice and establish the necessity of recommendation for colonoscopic evaluation in patients with increased background colonic uptake on PET. Accordingly, we analyzed the association between background FDG uptake grade on PET and the prevalence of CRA, which is a frequent precancerous lesion in the colon.

## Materials and Methods

### Study design and subjects

Patients' medical records from January 2006 to February 2015 in Ewha Womans University Mokdong Hospital, Seoul, Korea, were retrospectively reviewed. To evaluate the findings of PET scan and colonoscopy performed at the same period, this study included patients with gynecologic malignancy, whom our institute routinely performs both examinations for the initial baseline study. Patients with ovarian malignancies were excluded, because ovarian cancer itself or its peritoneal seeding can be overlapped or confused with colonic uptake. Patients with a history of infectious or inflammatory bowel disease, colonic malignancy, or metastatic colon lesion were excluded. We also excluded patients with age under 30 years old, incomplete medical records of colonoscopic or histopathologic findings, insufficient colonoscopy procedure, or poor bowel preparation.

### Collection of clinical data

For the medical record review, underlying diseases, age at diagnosis, gender, alcohol and smoking history, family history of colon cancer, height, and body weight were retrieved, and the laboratory findings within average of 6 days before or after ^18^F-FDG PET scan, including plasma glucose, serum triglyceride (TG), and total cholesterol, were also collected.

We calculated body mass index (BMI) as ‘body weight (kg) / height (m)^2^’ and a BMI value of 23 kg/m^2^ or greater was considered overweight in the Korean population. Glucose intolerance was defined as a fasting plasma glucose level of 100 mg/dL or higher, hypertriglyceridemia as a serum TG level of 150 mg/dL or higher, and hypercholesterolemia as a serum total cholesterol level of 200 mg/dL or higher.

### ^18^F-FDG PET/CT and image analysis

All patients were evaluated with ^18^F-FDG PET (103 patients) or PET/CT (138 patients). Before the ^18^F-FDG injection, patients fasted at least 6 hours and blood glucose level was confirmed to be < 140 mg/dL. The injected dose of ^18^F-FDG was 5.18 MBq/kg. After the ^18^F-FDG injection, patients were strictly instructed to rest for one hour. For ^18^F-FDG PET, a transmission scan for attenuation correction was obtained using the point source of ^137^Cs, and then followed by an emission scan, using an Allegro PET scanner (Philips-ADAC Medical Systems, Cleveland, OH). The emission scan was acquired for 3 minutes per bed position with 3D mode and reconstructed using a 3D OSEM iterative algorithm (4 iterations and 8 subsets).

For ^18^F-FDG PET/CT, a low-dose CT for attenuation correction was obtained first, using loss reduction software (CARE Dose, Siemens Medical Solutions, Erlangen, Germany) without any contrast agent, after which an emission PET scan was performed from the skull base to the thigh, using a dedicated PET/CT (Biograph mCT, Siemens Medical Solutions, Erlangen, Germany). The emission scan was acquired for 2 minutes per bed position with 3D mode and reconstructed using a 3D OSEM iterative algorithm (2 iterations and 21 subsets) with time of flight (TOF) and point spread function (PSF).

**Fig 1:**
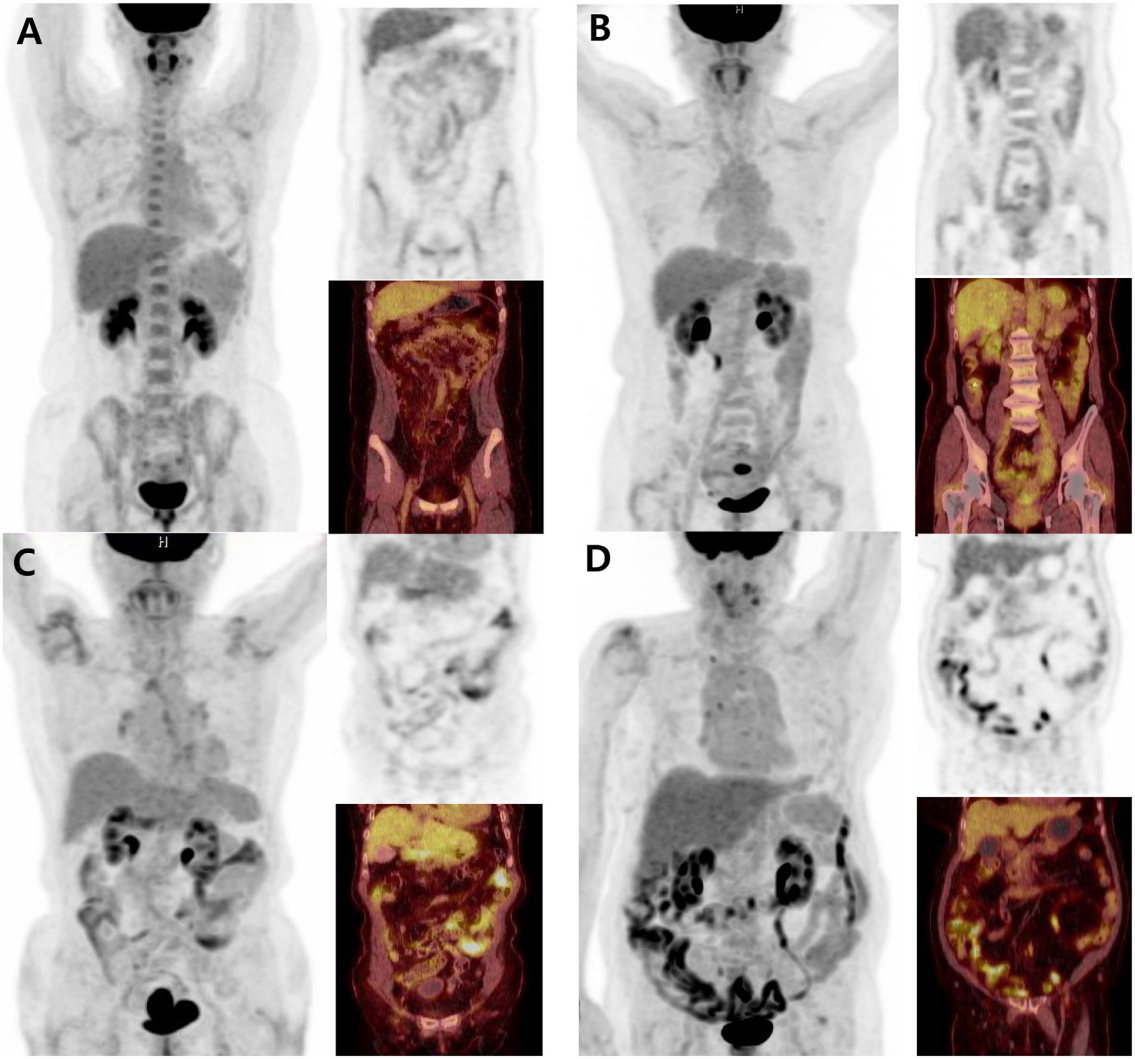
(A) The representative case of grade 1 background colonic ^18^F-FDG uptake on PET/CT scan (TB SUV_*max*_ of 1.9) is shown. (B) The representative case of grade 2 background colonic uptake on PET/CT scan (TB SUV_*max*_ of 2.0) is shown. (C) The representative case of grade 3 background colonic uptake on PET/CT scan (TB SUV_*max*_ of 3.4) is shown. (D) The representative case of grade 4 background colonic uptake on PET/CT scan (TB SUV_*max*_ of 5.3) is shown.

For qualitative and quantitative evaluation of background colonic uptake, the degree of non-focal diffuse uptake of colon was evaluated to minimize the effect by focal colonic adenoma. Background colonic ^18^F-FDG uptake was visually graded by two nuclear medicine specialists using a four-point scale; grade 1: background colonic ^18^F-FDG uptake of the whole colon is lower than that of the liver, grade 2: background colonic ^18^F-FDG uptake of at least one segment is equal to that of the liver, grade 3: background colonic ^18^F-FDG uptake of at least one segment is higher than that of the liver, grade 4: background colonic ^18^F-FDG uptake of almost all of the large intestine is higher than that of the liver (Fig 1). Grading was performed according to a consensus reached by the two readers, who were strictly blinded to other clinical information. For the quantitative analysis, the maximal standardized uptake value (SUV_*max*_) in 7 different bowel segments (i.e., duodenum, jejunum, ileum, cecum, hepatic flexure, splenic flexure, and descending colon-sigmoid junction) were measured after placement of a three-dimensional volume of interest (VOI) [11]. Then, the SUV_*max*_ of the 7 bowel segments were averaged to obtain the total bowel (TB) SUV_*max*_, which was used for further statistical analysis.

### Colonoscopy and diagnosis

All patients maintained a fasting state over 8 hours and bowel preparation was performed with polyethylene glycol before undergoing full colonoscopy. Electronic high resolution colonoscopy (CF-H260AL or CF-Q260AL, Olympus, Tokyo, Japan) was used for the examination. The colon was thoroughly observed from cecum to rectum, with withdrawal time more than 6 minutes. The grade of bowel preparation was classified into five levels according to the Aronchick scale (excellent, good, fair, poor, and inadequate) [12]. Adequate bowel preparation was defined as ‘fair’ or higher.

Six experienced endoscopists who had previously carried out colonoscopy more than 500 cases performed all colonoscopies, and recorded the size, location, and number of any colorectal neoplasm (CRN) that was identified. After removal of the CRN, histopathologic assessments were carried out by experienced pathologists. Inflammatory and hyperplastic polyps were classified as benign disease, as distinguished from CRA and CRC. Advanced CRA was defined as a CRA greater than 1 cm in diameter, CRA with high-grade dysplasia or containing a villous component.

### Statistical analysis

For comparison of clinical factors between the group with CRA and the group without CRA (non-CRA), the Chi-square test or Fisher’s exact test was performed for categorical variables, and the Student *t* test was used for continuous variables. Multivariate logistic regression analysis was performed to determine independent factors for the presence of CRA. The inter-observer agreement for visual grading of background colonic ^18^F-FDG uptake on PET was analyzed using kappa statistics. The correlation between visually classified background colonic ^18^F-FDG uptake grade and TB SUV_*max*_ on PET was analyzed using Spearman’s correlation. All statistical analyses were performed using SPSS version 22.0 (SPSS Inc., Chicago, IL, USA) and a value of *p* < 0.05 was considered statistically significant.

### Ethics statement

This study was approved by the institutional review board of Ewha Womans University Mokdong Hospital (IRB file No. 2015-05-015). Written informed consent could not be given by participants because this study was conducted in a retrospective manner. However, patients’ records were anonymized and deidentified prior to analysis to protect their privacy.

## Results

### Baseline clinical factors of study subjects: colorectal adenoma vs. non-adenoma

**Fig 2:**
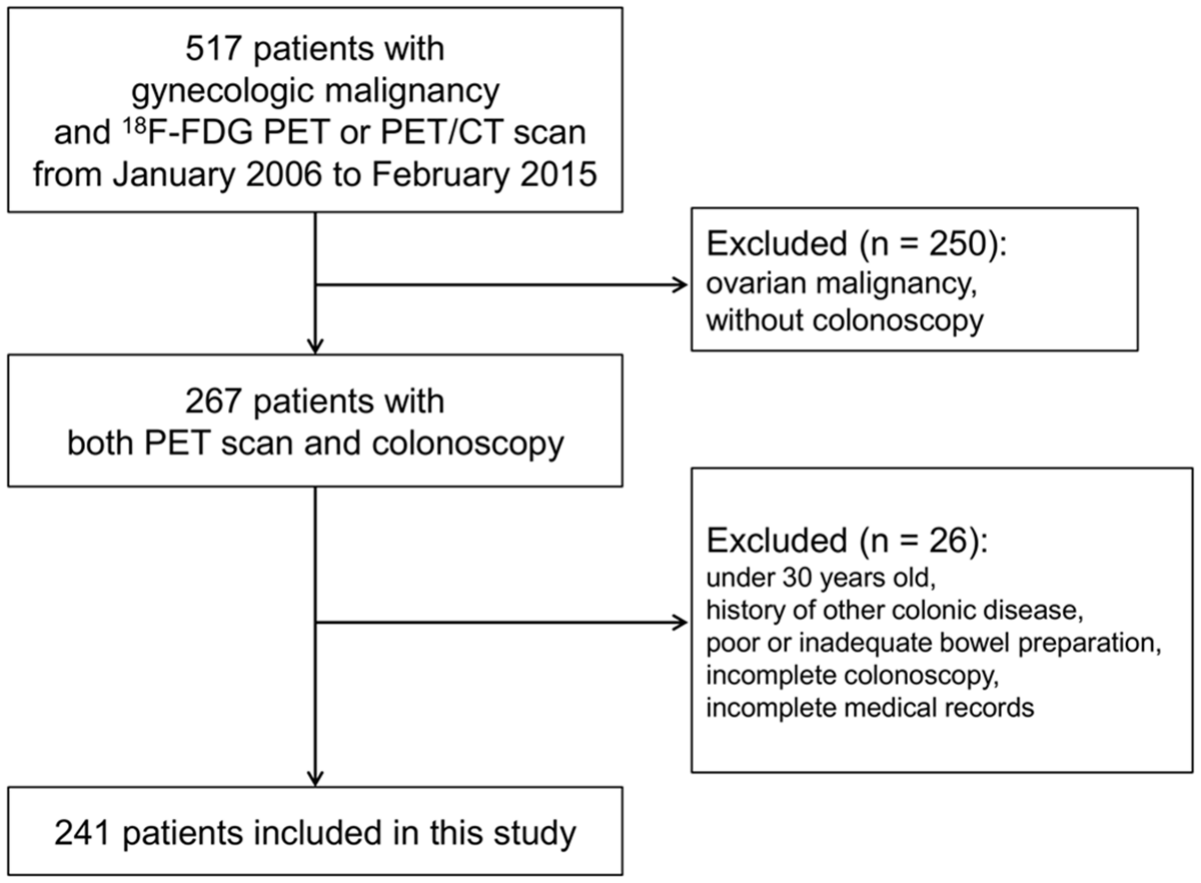
A flow diagram of patient selection in this study. FDG, fluoro-2-deoxy-D-glucose; PET, positron emission tomography; CT, computed tomography.

There were 517 patients who had received PET scan in the gynecology department between January 2006 and February 2015. Of these patients, 250 patients with ovarian malignancy or without colonoscopy were excluded. After excluding patients with age under 30 years old, a history of other colonic disease, poor or inadequate bowel preparation, incomplete colonoscopy, and no medical records of colonoscopy or biopsy findings (N = 26), a total of 241 patients were finally included in the present study (Fig 2).

In our study, the pathologic report of all patients with CRN showed adenoma or benign lesions, and there were no subjects with colorectal malignancy. Among a total of 241 study subjects, demographic data and clinical factors were compared between the CRA group and the non-CRA group (Table 1). The age at diagnosis was significantly higher in the CRA group than non-CRA group (61.1 ± 13.3 vs. 52.3 ± 12.2, *p* < 0.001). However, gender, alcohol use, smoking habits, and family history of CRC were not different between the two groups. Regarding the BMI, fasting plasma glucose, serum TG, and serum total cholesterol, only serum TG level was significantly higher in the CRA group than non-CRA group (116.8 ± 61.6 vs. 97.2 ± 49.9, *p* = 0.025), and other values did not show any considerable difference.

**Table 1:** Baseline Demographics and Clinical Factors of Study Subjects (N = 241).

	CRA (n = 45)	Non-CRA (n = 196)	*p*—value
Age at diagnosis (years)^a^	61.1 ± 13.1	52.3 ± 12.2	< 0.001
Alcohol use, n (%)			0.387^b^
Non drinker	45 (100)	188 (95.9)	
Social drinker	0 (0)	6 (3.1)	
Heavy drinker	0 (0)	2 (1.0)	
Cigarette smoking, n (%)			0.786^b^
Never smoker	44 (97.8)	189 (96.4)	
Ex-smoker	0 (0)	2 (1.0)	
Current smoker	1 (2.2)	5 (2.6)	
Family history of CRC, n (%)			0.512
Presence	1 (2.2)	2 (1.0)	
Absence	44 (97.8)	194 (99.0)	
Type of gynecologic disease, n (%)			0.664
Cervical cancer	28 (62.2)	134 (68.4)	
Endometrial cancer	17 (37.8)	59 (30.1)	
Others	0 (0)	3 (1.5)	
Body mass index (kg/m²)^a^	24.8 ± 3.2	23.9 ± 3.6	0.125
Fasting plasma glucose (mg/dL)^a^	102.7 ± 31.7	97.5 ± 19.9	0.293
Serum triglyceride (mg/dL)^a^	116.8 ±61.6	97.2 ± 49.9	0.025
Serum total cholesterol (mg/dL)^a^	180.9 ± 39.8	177.8 ± 37.1	0.619
Background colonic ^18^F-FDG uptake, n (%)			0.013^b^
Grade 1	9 (20.0)	63 (32.1)	
Grade 2	12 (26.7)	69 (35.2)	
Grade 3	22 (48.9)	60 (30.6)	
Grade 4	2 (4.4)	4 (2.0)	

CRA, colorectal adenoma; non-CRA, non-colorectal adenoma; CRC, colorectal cancer; ^18^F-FDG, ^18^F-fluoro-2-deoxy-D-glucose.

^a^Mean ± standard deviation

^b^ trend *p* value by linear-to-linear association analysis

According to the visual grade of background colonic ^18^F-FDG uptake on PET, the inter-observer agreement was almost perfect (Cohen’s weighted kappa value of 0.817): 29.9% of patients were classified as grade 1, 33.6% were grade 2, 34.0% were grade 3, and 2.5% were grade 4. For further statistical analysis, grade 1 and grade 2 were considered the low grade group (63.5%), while grade 3 and grade 4 were considered the high grade group (36.5%).

### Association of background colonic uptake grade on PET with the prevalence of colorectal adenoma

To identify the clinical factors associated with the prevalence of CRA, univariate analyses were performed for age at diagnosis, alcohol use, cigarette smoking, family history of CRC, BMI, fasting glucose, TG, total cholesterol, and background colonic uptake grade on PET scan (Table 2, univariate analysis). Consequently, the proportion of patients with older age at diagnosis (≥ 50 years, *p* = 0.034), overweight (BMI ≥ 23 kg/m², *p* = 0.010), and hypercholesterolemia (total cholesterol ≥ 200 mg/dL, *p* = 0.027) was significantly higher in the CRA group compared to the non-CRA group. In addition, high grade background colonic ^18^F-FDG uptake on PET was positively associated with the prevalence of CRA (*p* = 0.009). Multivariate analysis was performed adjusting all possible variables to identify independent predictors for the prevalence of CRA (Table 2, multivariate analysis). By multiple logistic regression model with adjusted other variables including age at diagnosis, alcohol use, cigarette smoking, family history of CRC, BMI, plasma glucose, serum TG, and total cholesterol, high grade background colonic ^18^F-FDG uptake was independently predictive of the possibility of having CRA. The odds of having CRA was 2.25 times higher in the high background colonic uptake grade group than low grade group (adjusted odds ratio (OR), 2.25, 95% confidence interval (CI), 1.13–4.49, *p* = 0.021).

**Table 2:** Association of background colonic uptake grade on PET with the prevalence of colorectal adenoma.

	Univariate analysis (Chi-square test)			Multivariate analysis (Logistic regression analysis)		
	CRA	Non-CRA	*p* value	aOR*	95% CI	*p* value
Age at diagnosis, n (%)			0.034			
< 50 years	12 (12.2)	86 (87.8)		1 (reference)		
≥ 50 years	33 (23.1)	110 (76.9)		1.83	0.83–4.01	0.134
Alcohol use, n (%)			0.168			
Non drinker	45 (19.3)	188 (80.7)		1 (reference)		
Social and heavy drinker	0 (0)	8 (100)		0	0	0.999
Cigarette smoking, n (%)			0.649			
Non smoker	44 (18.9)	189 (81.1)		1 (reference)		
Ex- or current smoker	1 (12.5)	7 (87.5)		0.54	0.05–5.38	0.595
Family history of CRC, n (%)			0.512			
Absence	44 (18.5)	194 (81.5)		1 (reference)		
Presence	1 (33.3)	2 (66.7)		4.28	0.34–53.40	0.259
Body mass index, n (%)			0.010			
< 23 kg/m²	11 (11.0)	89 (89.0)		1 (reference)		
≥ 23 kg/m²	34 (24.1)	107 (75.9)		1.91	0.88–4.15	0.101
Plasma glucose, n (%)			0.226			
< 100 mg/dL	28 (16.7)	140 (83.3)		1 (reference)		
≥ 100 mg/dL	17 (23.3)	56 (76.7)		1.17	0.56–2.42	0.676
Triglyceride, n (%)			0.067			
< 150 mg/dL	35 (16.9)	172 (83.1)		1 (reference)		
≥ 150 mg/dL	10 (30.3)	23 (69.7)		1.71	0.69–4.23	0.244
Total cholesterol, n (%)			0.027			
< 200 mg/dL	26 (15.2)	145 (84.8)		1 (reference)		
≥ 200 mg/dL	19 (27.5)	50 (72.5)		1.90	0.92–3.91	0.081
Background colonic ^18^F-FDG uptake, n (%)			0.009			
Low grade (1, 2)	21 (13.7)	132 (86.3)		1 (reference)		
High grade (3, 4)	24 (27.3)	64 (72.7)		2.25	1.13–4.49	0.021

CRA, colorectal adenoma; non-CRA, non-colorectal adenoma; aOR, adjusted odds ratio, CI, confidence interval; CRC, colorectal cancer; ^18^F-FDG, ^18^F-fluoro-2-deoxy-D-glucose.

*presence of colorectal adenoma as the dependent variable

We analyzed the prevalence of advanced and non-advanced CRAs according to the grade of background uptake; the prevalence of advanced CRA was not significantly different between low (Grade 1, 2) and high grade background uptake (Grade 3, 4) (*p* = 0.274). We also analyzed the number of CRAs according to the grade of background uptake; 3 or more CRAs was not significantly different than 1 or 2 CRAs (*p* = 0.578) (S1 Table).

### Patients with colorectal adenoma according to the grade of background colonic uptake on PET scan

**Fig 3:**
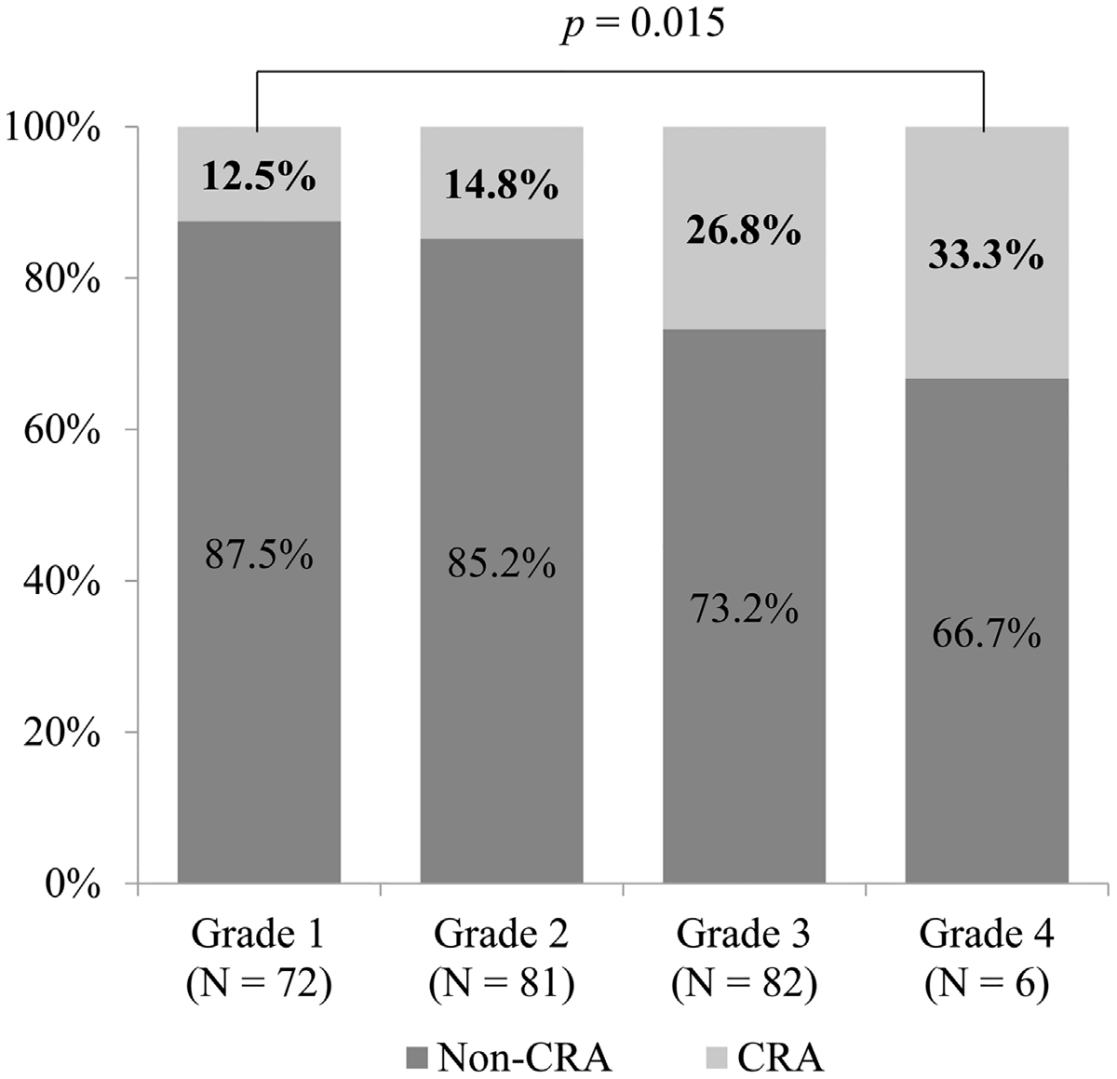
The proportion of patients with colorectal adenoma demonstrated rising trend with increasing grade of background colonic ^18^F-FDG uptake on PET scan (trend *p* = 0.015 by linear-by-linear association analysis). CRA, colorectal adenoma.

Percentages of patients with CRA according to the grade of background colonic ^18^F-FDG uptake on PET scan were analyzed (Fig 3). The proportion of patients with CRA was 12.5% in grade 1, 14.8% in grade 2, 26.8% in grade 3, and 33.3% in grade 4. Thus, the proportion of patients with CRA significantly increased as the grade of background colonic uptake became higher (trend *p* = 0.015 by linear-by-linear association analysis).

Out of a total of 241 subjects in this study, TB SUV_*max*_ values were calculated for the 138 patients who underwent PET/CT scan. In quantitative analysis, TB SUV_*max*_ was 2.5 ± 0.68 in the CRA group, and 2.3 ± 0.55 in the non-CRA group. TB SUV_*max*_ was higher in the CRA group than non-CRA group, but it did not reach a statistical significance (*p* = 0.080). However, when the study subjects were classified according to the cut-off value of TB SUV_*max*_, statistical significance was observed in further analyses. A ROC curve was obtained regarding TB SUV_*max*_ values and the cut-off value was 2.25. Subsequently, a Chi-square test was performed to determine the proportion of patients with CRA among those with SUV_*max*_ > 2.25 (high SUV_*max*_ group, N = 63) compared with those with SUV_*max*_ ≤ 2.25 (low SUV_*max*_ group, N = 75). The proportion of CRA patients was significantly higher in the high SUV_*max*_ group than in the low SUV_*max*_ group (27.5% vs. 11.6%, *p* = 0.031). Additionally, the correlation between the visual grade of background colonic 18F-FDG uptake and TB SUV_*max*_ was also statistically significant (*Spearman’s rho* = 0.719, *p* < 0.001).

By multiple logistic regression model with age at diagnosis, alcohol use, cigarette smoking, family history of CRC, BMI, plasma glucose, serum TG, and total cholesterol, and TB SUV_*max*_, high TB SUV_*max*_ (> 2.25) was independently predictive of the possibility of having CRA (aOR 2.89, *p* = 0.038) (S2 Table).

## Discussion

The purpose of this study was to identify the clinical significance of background colonic ^18^F-FDG uptake on PET scan. The results demonstrated that background colonic ^18^F-FDG uptake on PET was an independent factor associated with the prevalence of CRA, and that the proportion of patients with CRA became higher with an increase in the grade of background colonic uptake in a dose-dependent manner.

A recent study suggested that background intestinal ^18^F-FDG uptake on PET can be associated with gut flora [10]. In patients with nongastrointestinal lymphoma who had PET/CT scan after taking rifaximin, a nonabsorbed antibiotic, for 2 days, cecal SUV_*max*_ and the proportion of colonic ^18^F-FDG uptake greater than grade 1 during the post-rifaximin scan were significantly lower than those during the pre-rifaximin scan [10]. Considering several reports demonstrating that rifaximin changes the gut microbiota [13,14], the decrease in background intestinal ^18^F-FDG uptake could be explained by alteration of the gut flora. In addition, it has been reported that the microbiota of the colon in patients with and without CRC or CRA is different [15–17]. Taken together, we assumed that the contribution of gut flora may explain the significant association between background intestinal ^18^F-FDG uptake and the prevalence of CRA in the present study. However, this was an observational study to identify the association between background colonic FDG uptake and CRA. Thus, further translational and experimental studies, including metagenomic analyses, are necessary to clarify the underlying mechanism.

On the other hand, obesity and hypertriglyceridemia can be another underlying mechanism for our results. Yoon et al. [11] showed that obesity and hypertriglyceridemia may be related to high background intestinal ^18^F-FDG uptake on PET scan. In non-diabetic and non-hypertensive breast cancer patients, BMI and TG levels were higher as the visual grade of background intestinal ^18^F-FDG uptake and TB SUV_*max*_ increased [11]. It is already known that obesity is related to the prevalence of CRA [18–25]. Obesity is closely related to hyperinsulinemia and insulin resistance, which is associated with an increase in insulin like growth factor (IGF) levels [26]. It was identified that high levels of insulin and IGFs can promote cancer development through insulin/IGF axis, and especially that IGF-1 plays an important role in the inhibition of apoptosis and promotion of cell-cycle progression [27–29]. In addition, some studies demonstrated that hypertriglyceridemia is significantly associated with an increase in the prevalence of CRA [23,30]. This association of hypertriglycemia with CRA can be explained by the insulin/IGF-1 pathway [31], oxidative stress [32], and proinflammatory cytokines such as tumor necrosis factor α and interleukin (IL)-6 [33]. In the correlation analysis in our study, BMI (*r* = 0.155, *p* = 0.016) and TG levels (*r* = 0.151, *p* = 0.019) showed weak positive correlations with background colonic FDG uptake. Considering the significant association between background FDG uptake and obesity/hypertriglyceridemia, it could be assumed that background intestinal FDG uptake may be associated with the prevalence of CRA. However, further observational and experimental studies are warranted to explain its underlying mechanisms and obtain direct evidence.

There are several limitations in this study. First, it was a retrospective study reviewing medical records of subjects who had received both PET scan and colonoscopy. There were some missing data in the baseline characteristics including underlying disease and laboratory results. Among them, some medication may have influenced the physiologic intestinal ^18^F-FDG uptake. Particularly, the effect of metformin intake in diabetic patients has been reported by several previous studies [8,9]. According to our reviewing medical records, 4 diabetic patients were prescribed metformin among the 241 patients. A Fisher’s exact test did not show a significant difference in background colonic uptake based on metformin intake (*p* = 0.624). However, the small sample size of the metformin intake group could contribute to the statistical insignificance. If the retrospective chart review missed the exact number of diabetic patients with metformin intake, it could be one of limitations of this study. Second, how to exclude uptake by CRA from background colonic ^18^F-FDG uptake on PET scan could raise a possibility of bias. Though CRA can show variable degree of FDG uptake, the most common uptake pattern of CRA is a focal nodular uptake [5,34]. Thus, we evaluated non-focal diffuse uptake of colon to minimize the effect by CRA. For the low grade group, we believe that most of CRA uptake can be avoided by evaluating non-focal diffuse colonic uptake. However, in the high background colonic uptake group, it is difficult to discriminate focal colonic uptake from background colonic uptake. Even if the discrimination of focal colonic uptake is impossible due to high background colonic uptake, and if the measurement was performed at the site of colonic adenoma, diffuse background colonic uptake would be high enough to mask focal uptake of CRA. Third, the colonoscopies were performed by several endoscopists, not by a single person. This may have acted as a bias because the adenoma detection rate may differ according to the level of competence and experience of the endoscopists. However, to minimize this limitation, this study included only examinations performed by experienced endoscopists, each of whom had performed more than 150 colonoscopic examinations. Furthermore, only examinations with a withdrawal time of more than 6 minutes and a level of bowel preparation 'fair' or higher on the Aronchick scale were included to improve the quality of the study.

## Conclusion

This study shows that a high grade of background colonic ^18^F-FDG uptake is significantly associated with a high possibility of having CRA. In addition, as the grade of background colonic ^18^F-FDG uptake increases, the proportion of CRA increases in a dose-dependent manner. Therefore, although patients undergo PET scans for many other indications, physicians should be alert to the possibility of finding high background colonic FDG uptake and considering its implications.

## Supporting Information

S1 Table Association of background colonic FDG uptake with the number or grade of colorectal adenoma (CRA). The prevalence of advanced CRA and the number of CRA according to the grade of background uptake was analyzed.
(DOCX)

S2 Table Association of total bowel SUVmax on PET with the prevalence of colorectal adenoma. Multiple logistic regression analysis was performed with the presence of colorectal adenoma as the dependent variable.
(DOCX)
