# Supplementary material for: Background Colonic 18F-Fluoro-2-Deoxy-D-Glucose Uptake on Positron Emission Tomography Is Associated with the Presence of Colorectal Adenoma
Source: PLoS One. 2016 Aug 10;11(8):e0160886. doi: 10.1371/journal.pone.0160886 (PMC4979890; doi:10.1371/journal.pone.0160886)
Supplement: S1 Table — The prevalence of advanced CRA and the number of CRA according to the grade of background uptake was analyzed. (DOCX) [file pone.0160886.s001.docx]

**Supporting Table 1. Association of background colonic FDG uptake with the number or grade of colorectal adenoma (CRA).** The prevalence of advanced CRA and the number of CRA according to the grade of background uptake was analyzed.

|  | Low background colonic uptake grade (n = 153) | High background colonic uptake grade (n = 88) | *p* - value |
| --- | --- | --- | --- |
| Presence of advanced CRA, n (%) |  |  | 0.274 |
| Non-advanced CRA | 16 (76.2) | 21 (87.5) |  |
| Advanced CRA | 5 (23.8) | 3 (12.5) |  |
| Number of CRA, n (%) |  |  | 0.578 |
| 1 - 2 | 18 (85.7) | 20 (83.3) |  |
| ≥ 3 | 3 (14.3) | 4 (16.7) |  |

CRA, colorectal adenoma
