# Supplementary material for: Background Colonic 18F-Fluoro-2-Deoxy-D-Glucose Uptake on Positron Emission Tomography Is Associated with the Presence of Colorectal Adenoma
Source: PLoS One. 2016 Aug 10;11(8):e0160886. doi: 10.1371/journal.pone.0160886 (PMC4979890; doi:10.1371/journal.pone.0160886)
Supplement: S2 Table — Multiple logistic regression analysis was performed with the presence of colorectal adenoma as the dependent variable. (DOCX) [file pone.0160886.s002.docx]

**Supporting Table 2. Association of total bowel SUV*_max_* on PET with the prevalence of colorectal adenoma.** Multiple logistic regression analysis was performed with the presence of colorectal adenoma as the dependent variable.

|  | Multivariate analysis (Logistic regression analysis) | | |
| --- | --- | --- | --- |
|  | aOR^*^ | 95% CI | *p* value |
| Age at diagnosis, n (%) |  |  |  |
| < 50 years | 1 (reference) |  |  |
| ≥ 50 years | 0.97 | 0.37 - 2.55 | 0.953 |
| Alcohol use, n (%) |  |  |  |
| Non drinker | 1 (reference) |  |  |
| Social and heavy drinker | 0 | 0 | 0.999 |
| Cigarette smoking, n (%) |  |  |  |
| Non smoker | 1 (reference) |  |  |
| Ex- or current smoker | 0 | 0 | 0.999 |
| Family history of CRC, n (%) |  |  |  |
| Absence | 1 (reference) |  |  |
| Presence | 3.87 | 0.28 – 53.90 | 0.314 |
| Body mass index, n (%) |  |  |  |
| < 23 kg/m² | 1 (reference) |  |  |
| ≥ 23 kg/m² | 1.10 | 0.42 – 2.85 | 0.846 |
| Plasma glucose, n (%) |  |  |  |
| < 100 mg/dL | 1 (reference) |  |  |
| ≥ 100 mg/dL | 1.17 | 0.46 – 3.01 | 0.741 |
| Triglyceride, n (%) |  |  |  |
| < 150 mg/dL | 1 (reference) |  |  |
| ≥ 150 mg/dL | 1.72 | 0.44 – 6.69 | 0.432 |
| Total cholesterol, n (%) |  |  |  |
| < 200 mg/dL | 1 (reference) |  |  |
| ≥ 200 mg/dL | 1.25 | 0.48 – 3.29 | 0.651 |
| TB SUV_max_ |  |  |  |
| ≤ 2.25 | 1 (reference) |  |  |
| > 2.25 | 2.89 | 1.06 – 7.88 | 0.038 |

aOR, adjusted odds ratio, CI, confidence interval; CRC, colorectal cancer; TB, total bowel; SUV_max_, maximal standardized uptake value.

*presence of colorectal adenoma as the dependent variable.
